# Genetic Variants Associated with Hyperandrogenemia in PCOS Pathophysiology

**DOI:** 10.1155/2018/7624932

**Published:** 2018-02-18

**Authors:** Roshan Dadachanji, Nuzhat Shaikh, Srabani Mukherjee

**Affiliations:** Department of Molecular Endocrinology, National Institute for Research in Reproductive Health, J.M. Street, Parel, Mumbai 400012, India

## Abstract

Polycystic ovary syndrome is a multifactorial endocrine disorder whose pathophysiology baffles many researchers till today. This syndrome is typically characterized by anovulatory cycles and infertility, altered gonadotropin levels, obesity, and bulky multifollicular ovaries on ultrasound. Hyperandrogenism and insulin resistance are hallmark features of its complex pathophysiology. Hyperandrogenemia is a salient feature of PCOS and a major contributor to cosmetic anomalies including hirsutism, acne, and male pattern alopecia in affected women. Increased androgen levels may be intrinsic or aggravated by preexisting insulin resistance in women with PCOS. Studies have reported augmented ovarian steroidogenesis patterns attributed mainly to theca cell hypertrophy and altered expression of key enzymes in the steroidogenic pathway. Candidate gene studies have been performed in order to delineate the association of polymorphisms in genes, which encode enzymes in the intricate cascade of steroidogenesis or modulate the levels and action of circulating androgens, with risk of PCOS development and its related traits. However, inconsistent findings have impacted the emergence of a unanimously accepted genetic marker for PCOS susceptibility. In the current review, we have summarized the influence of polymorphisms in important androgen related genes in governing genetic predisposition to PCOS and its related metabolic and reproductive traits.

## 1. Introduction

Polycystic ovary syndrome affects scores of women worldwide with a prevalence of nearly 6–10% of premenopausal women [1]. Typical features of PCOS comprise distorted gonadotropin ratios, chronic anovulation, and subsequently irregular menstrual cycles, insulin resistance, increased androgen levels, and appearance of polycystic ovarian morphology upon ultrasound imaging [2]. Besides reproductive anomalies, these women are at an increased risk of developing type II diabetes, metabolic syndrome, and cardiovascular diseases (CVD), with subclinical markers being detected at earlier ages. While a clear-cut origin of PCOS has not emerged to explain its underlying pathophysiology, androgen excess and insulin resistance are reported to be the pivotal pathogenic drivers which extend reproductive, metabolic, and cosmetic consequences to affected women. The ovary remains the primary source of hyperandrogenism in women with PCOS which is mainly attributed to thecal cell hyperplasia leading to intense ovarian steroidogenesis. Evidence suggests that hyperandrogenism is an important factor in promoting anovulation due to follicular arrest [3] and high androgen level has been linked to reduced oocyte developmental competence and maturation rates. Further testosterone has been correlated with fertilization rates, embryo development, and miscarriage rates in women with PCOS [4]. Adrenal androgen excess has been reported in 20–30% of women with PCOS possibly due to defects in cortisol metabolism or common steroid pathway biosynthesis enzymes [5]. Apart from modifying reproductive outcomes, hyperandrogenemia also predicts severity of cardiometabolic profiles and CVD risk [6]. A recent meta-analysis has accentuated that hyperandrogenemia unfavourably influences the incidence of dyslipidemia, indices of insulin resistance, and metabolic syndrome risk [7]. The possibility of a genetic basis of hyperandrogenemia in PCOS has been recognized early [8] and candidate gene studies investigating the association of genes involved in androgen synthesis and action have strengthened this concept further [9, 10]. In the present review, we have outlined studies detailing the association of polymorphisms in these genes with PCOS susceptibility and its related traits.

## 2. General Steroid Metabolism

Ovary, the chief organ of interest is endowed with important functions of maintaining the female reproductive physiology. These include timely development of ovarian follicles and production of mature oocytes as well as the steroid hormones synthesis [11]. Steroidogenesis comprises processes by which the precursor cholesterol is converted to biologically active steroid hormones. Steroidogenic enzymes are responsible for the biosynthesis of various steroid hormones including glucocorticoids, mineralocorticoids, progestins, androgens, and estrogens. They consist of several specific cytochrome P450 enzymes (CYPs), hydroxysteroid dehydrogenases (HSDs), and steroid reductases [12]. De novo synthesis of all steroid hormones starts with the conversion of cholesterol to pregnenolone by CYP11A (cholesterol side-chain cleavage) [13]. CYP11A is bound to the inner membrane of the mitochondrion and is found in all steroid producing organs and tissues [12]. Pregnenolone is converted to progesterone by 3*β*-hydroxysteroid dehydrogenase (3*β*-HSD), found in both mitochondria and smooth endoplasmic reticulum. 3*β*-HSD is widely distributed in steroidogenic and nonsteroidogenic tissues and consists of two isoenzymes, which are regulated in a tissue-specific manner [14–17]. The type 2 3*β*-HSD is predominantly expressed in steroidogenic tissues such as adrenal, testis, and ovary, whereas type 1 is found in placenta and in nonsteroidogenic tissues such as liver, kidney, and skin. Pregnenolone and progesterone form the precursors for all other steroid hormones.

In the ovary, steroidogenesis is a well-regulated process governed by the gonadotropins and signaling mechanisms occurring in the ovarian cells. Androgen synthesis predominantly takes place in thecal cells which have LH receptors and subsequent signaling and activation of CYP17 enzyme convert pregnenolone and progesterone to dehydroepiandrosterone (DHEA) and androstenedione, respectively. These androgens are further acted upon by CYP19 aromatase enzymes present in the FSH stimulated granulosa cells to estrogens which are essential for normal physiological functions of the human ovary.

## 3. Hyperandrogenemia and PCOS

The most common clinical manifestation of hyperandrogenism in women is hirsutism and excessive terminal hair growth in androgen-dependent areas of the body. Other clinical manifestations of hyperandrogenism include acne vulgaris, weight gain, menstrual irregularities, and acanthosis nigricans [1]. Hyperandrogenemia has been the common feature included in all three mainly proposed and employed diagnostic criteria put forward by the National Institute of Health in 1990, consensus criteria by the American Society for Reproductive Medicine (ASRM) and the European Society of Human Reproduction and Embryology (ESHRE) at Rotterdam in 2003, and more recently the Androgen Excess Society in 2006, which has asserted the inclusion of presence of clinical and/or biochemical hyperandrogenism to be imperative in diagnosis of PCOS. A controversial opinion regarding the inclusion of hyperandrogenism was debated at the 2012 joint meeting of ASRM and ESHRE whereupon it was noted that it was a significant predictor for diagnosis and prognosis of the syndrome and its accompanying metabolic maladies, thus forming an important criterion for inclusion into multicentric studies in PCOS [18].

PCOS is now considered as a disorder of androgen excess [19, 20]. In women with PCOS, there is increased gonadotropin-releasing hormone (GnRH) pulse frequency which favours increased LH secretion over that of follicle stimulating hormone (FSH) [21]. Under control of high pulsatile release of LH, the theca cells upregulate the expression of steroidogenic acute regulatory protein (StAR), P450 side-chain cleavage (P450scc), 3*β*-hydroxysteroid dehydrogenase (3*β*-HSD), and cytochrome P450c17 (CYP17) and increase steroidogenic activity in the theca cells [22], thereby producing androstenedione. This action is further enhanced in a synergistic fashion by the high levels of insulin commonly observed in PCOS women. Androstenedione is then converted by aromatase to estrogen in the granulosa cells under the influence of pituitary FSH. However, there is relative deficit in FSH secretion which often results in impaired and arrested follicular development and reduced aromatase activity, thereby resulting in excess androgen accumulation and hyperandrogenemia in PCOS women.

Polycystic ovaries typically consist of numerous follicles arrested primarily in the preantral and antral stages with thecal hyperplasia and follicular fluid accumulation subsequently forming cyst-like structures which line the periphery of the ovary giving it a string of pearls-like appearance. Increased ovarian stromal volume along with many fluid filled follicles make these ovaries enlarged, a common morphological feature observed in PCOS women. In addition to thickened thecal layers, these follicles show increased steroidogenic activity. Insulin resistance, another major player in PCOS pathophysiology, intensifies the steroid inducing action of LH and indirectly increases LH pulse amplitude and progressively worsens this hyperandrogenemia. Insulin may also act directly via the insulin receptors on the ovary to augment ovarian steroidogenesis [23] and may also stimulate P450c17*α* activity in ovary and adrenal glands of PCOS women [24]. Insulin indirectly exacerbates hyperandrogenemia by reducing hepatic biosynthesis of sex hormone binding globulin and increasing the free and bioavailable testosterone levels. This creates a precarious physiologic environment of hormonal imbalance promoted by a sequence of hyperinsulinemia followed by hyperandrogenemia [23]. Coupled with lowered aromatase activity and diminished conversion of testosterone to estrogen, the circulating androgen pool continues to grow. Long-term cultures of theca and granulosa cells demonstrated significantly increased enzymatic activities of P450c17*α* and 3*β*HSD in PCOS theca cells with subsequent increased synthesis of testosterone precursors compared to normal cells [25, 26]. Follicular hyperandrogenemia induces marked changes in methylation status of essential genes for reproduction and development such as* PPARγ*,* HDAC3*, and* NCOR1* [27]. Increased activity of both 17 and 20 lyase in the Δ4 pathway and 3*β*-hydroxysteroid dehydrogenase II combined with low aromatase activity was documented in hyperandrogenic PCOS women. Thus, the heterogeneity in androgenic phenotype may be attributed to differential activity of important enzymes in the steroidogenic pathway [28].

While the metabolic derangements of PCOS have mainly been attributed to the insulin resistance and obesity frequently present in these women, increased abdominal adiposity develops as a consequence of hyperandrogenemia. Sensitive indicators of hyperandrogenism include total and free testosterone and androstenedione levels, and free androgen index (FAI) which are capable of predicting phenotype heterogeneity and severity [29]. Recently use of mass spectrometry based techniques such as liquid chromatography- and gas chromatography-MS/MS for serum and urinary steroid hormone profiling have led to more accurate measurements of androgen levels in women with PCOS and have been hailed as the gold standard for testosterone assay by the Endocrine Society [30]. These techniques have enabled researchers to accurately measure low concentrations of testosterone (<5 nmol/l) and prevent inaccurate measurement due to cross-reactivity with DHEAS as seen in immunoassay methods [31]. This has led to assignment of lower cut-off values for total and free testosterone [32], thereby improving diagnosis and subclassification of PCOS women [33]. However hirsutism scores do not necessarily correlate with steroid concentrations measured in affected women [29]. Further, PCOS women showing biochemical androgen excess are more prone to reduced insulin sensitivity and dysglycemia [34], number of menstrual cycles in a year, elevated cardiovascular disease markers, dyslipidemia, and heightened predisposition to metabolic syndrome [35].

Experimental evidence strongly implicates the role of hyperandrogenic intrauterine hormonal milieu in influencing the development of PCOS-like reproductive and metabolic features in monkey and sheep animal models. Prenatally androgenized ewes showed early increase in LH secretion, coupled with progressive loss in reproductive cyclicity and ovulatory failure [36], increased number of primary follicles [37], and persistent follicular cysts [38] along with upregulation of steroidogenic genes such as* StAR*,* CYP11A*, and* CYP17* in thecal cells of female offspring [39]. Similarly, female rhesus monkeys prenatally exposed to testosterone demonstrate LH hypersecretion, anovulation, and polyfollicular ovaries with increased follicular recruitment with impaired oocyte developmental competence [40]. Moreover, maternal or fetal exposure to high doses of androgens has been reported to alter pancreatic morphology, particularly beta cell development, which may contribute to insulin resistance and consequent hyperinsulinemia in rhesus monkeys and sheep models [41, 42]. Adrenal androgen excess has also been reported in prenatally androgenized nonhuman primate models for PCOS as indicated by elevated basal circulating levels of DHEA and DHEAS. Furthermore, treatment with thiazolidinedione-based insulin sensitizers ameliorates adrenal steroidogenesis along with reducing insulin resistance [43]. Women with PCOS were more likely to give birth to small for gestational age infants which has been associated with increased maternal testosterone levels [44]. Findings of high AMH levels in daughters born to women with PCOS suggest altered follicular development from an early age [45]. Familial prevalence of PCOS and its associated phenotypes provides evidence of possible maternal transmission and genetic inheritance of this disorder [10]. Maternal heritability had significant effects on the prevalence of fasting dysglycemia in women with PCOS [46]. These studies support prenatal activation and fetal programming which passes PCOS-like traits in subsequent generations. Xita et al. have elegantly hypothesized that exposure to androgen excess encourages fetal programming of PCOS by altering phenotypic expression of reproductive and metabolic tissues and results in altered differentiation of thecal cells, LH hypersecretion, and male-type fat distribution in female offspring. Moreover genotypes related to regulating androgen levels, activity and bioavailability in PCOS mothers can modulate the extent of androgen exposure in utero [47]. Epigenetic changes and subsequently altered gene expression and maternal nutrition have also been found to influence fetal programming [47–49]. On the other hand, it was also shown that increased maternal androgen levels may not induce PCOS in female fetus provided normal placental aromatization activity is maintained [49]. Altogether, these findings highlight the role of hyperandrogenism in critical windows of fetal development in modifying PCOS susceptibility.

**Figure 1:**
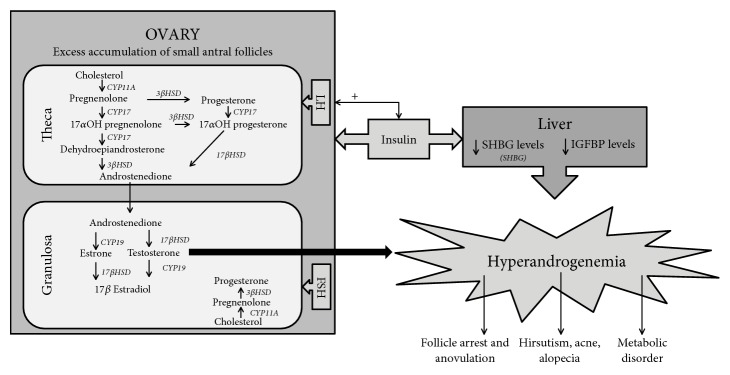
Overview of pathophysiology of PCOS. Androgen biosynthesis is a well-orchestrated process occurring in the ovary mediated by an enzymatic cascade under stimulation by pituitary LH. In PCOS, accumulation of small antral follicles with thecal hyperplasia along with overexpression of steroidogenic enzymes results in elevated testosterone levels. In contrast, downregulation of aromatase enzymes decreases testosterone to estradiol conversion, leading to release of large amounts of circulating testosterone. In addition, women with PCOS display insulin resistance coupled with compensatory hyperinsulinemia. Insulin acts directly on the ovary, via its receptors, as well as synergistically with LH to enhance androgen production. On the other hand, insulin acts indirectly via decreasing hepatic biosynthesis of sex hormone binding globulin, thereby raising biologically available testosterone levels. The hyperandrogenic phenotype is typically characterized by arrest in folliculogenesis and consequent anovulatory infertility and cosmetic problems such as hirsutism, acne, and androgenic alopecia. It also contributes to increased incidence of metabolic disorders including insulin resistance, dyslipidemia, metabolic syndrome, and cardiovascular disease.

PCOS is also thus regarded as a form of functional ovarian hyperandrogenism, where all the above-mentioned aberrations contribute towards not only the reproductive dysfunctions but also the metabolic anomalies observed in these women (Figure 1).

## 4. Genetics of PCOS

PCOS has a strong genetic component as evidenced by clustering of PCOS in families as well as PCOS-like features in both male and female relatives of affected women [1]. Approaches such as twin studies and linkage studies have been employed in order to decipher the contribution of heritability in this multifactorial disorder. Linkage studies of 37 candidate genes predicted strong association of follistatin and nominal association of* CYP11A1* gene in affected siblings with hyperandrogenemia and PCOS related traits. This same study established strong genetic association of* D19S884* allelic marker near* INSR* gene with PCOS, by transmission disequilibrium test [50]. Association studies of candidate genes involved in pathways related to the etiology of the syndrome and its associated anomalies have garnered interest from the research community to try and pinpoint the significance of genetic predisposition in manifestation of this syndrome [10]. Polymorphisms of genes involved in pathways including insulin signaling, gonadotropin regulation, chronic inflammation, and energy homeostasis have been studied [10]; however, the exact role of these susceptibility genes has not yet been established. Single nucleotide polymorphisms (SNPs) reveal functional changes due to the fact that amino acid variations or modulation of gene expression and candidate gene approaches are helpful in deciphering the impact of differential frequency distribution in healthy and diseased population. Simultaneously, while candidate gene approaches have been studied in relatively smaller populations, genome-wide association studies have revolutionized the study of PCOS genetics. Previously we have reviewed the genes involved in insulin action and regulation with PCOS susceptibility and related traits [51]. Given the importance of androgens in female reproductive health and PCOS development, in the current review, we will be concentrating on polymorphisms in genes involved in androgen synthesis, action, and bioavailability.

*CYP11A1 Gene*.* CYP11A1* on 15q23-24 encodes the enzyme P450 cholesterol side-chain cleavage that catalyzes the rate limiting step of ovarian steroidogenesis, that is, the conversion of cholesterol to pregnenolone [52, 53]. Theca cells derived from PCOS ovaries and propagated in long-term culture demonstrate increased CYP11A expression compared to normal theca cells [54, 55]. An early linkage study carried out in 20 families showed involvement of* CYP11A* locus in PCOS development and subsequently association of 5′UTR (TTTTA)_n_ pentanucleotide repeats in hirsute PCOS women [56]. Positive association of pentanucleotide repeat alleles with PCOS susceptibility were confirmed subsequently in women from United States [57], South India [58], and Greece [59] and nominally in women from United Kingdom [60]. Wang et al. have demonstrated that different allele combinations may increase or decrease the risk of PCOS in Chinese women [61]. In contrast to earlier findings, no association was reported in Spanish [62], Chinese [63, 64], Argentinian [65], Indian [53], and Czech [66] women with PCOS. A recent meta-analysis confirmed strong association of this (TTTTA)_n_ repeat polymorphism of* CYP11A* with increased risk of PCOS in Caucasian population [67]. Furthermore, another meta-analysis indicated that carriers of 4 repeats had increased risk considering the recessive model while carriers of 6 repeats showed decreased risk of PCOS considering the dominant model [68]. Conflicting reports regarding the association of these pentanucleotide repeats with PCOS related traits are available. Increased testosterone levels have been reported in carriers of short alleles in women with PCOS [53, 59] while no effect of allele dose was seen on CYP11A transcription [57] or serum androgen levels in another studies [57, 60, 63]. What is more this repeat polymorphism shows significant relationship with metabolic traits including obesity [61], higher waist-hip ratio, decreased AUC glucose values [64], alleviated dyslipidemia [66], and decreased FSH levels [66]. Another polymorphism, rs4077582, showed significant association in Chinese women with PCOS [69, 70] as well as altered testosterone and LH levels [70]. One more polymorphism, namely, rs11632698, showed both positive [69] and negative [70] association with PCOS risk in Chinese women. Together, these studies imply* CYP11A* to be a promising genetic biomarker for PCOS.

*CYP17 Gene*. The* CYP17* gene at 10q24.3 encodes cytochrome P450 enzyme with 17-hydroxylase activity, which converts pregnenolone and progesterone into 17-hydroxypregnenolone and 17-hydroxyprogesterone, respectively. The 17,20-lyase activity subsequently converts these steroids to dehydroepiandrosterone (DHEA) and 4-androstenedione [10]. The vast majority of studies have focused on a widely studied polymorphism at −34 position (−34 T/C) in the promoter, which creates an additional Sp1 transcription factor binding site, thereby regulating expression of CYP17 and consequently androgen levels [71]. In 1994, Carey et al. showed significant association of this polymorphism with PCO and male pattern baldness in a family-based study [72]; however, these findings were not persistent when they increased the sample size [73]. On similar lines, this polymorphism was not found to be a significant factor for PCOS development in British [74], Slovenian [75], Polish [76], American [77, 78], Korean [79], Chilean [80], Chinese [81], Thai [82], and Indian [83] women with PCOS or even in Turkish adolescents [84]. In contrast, Indian women with PCOS showed significantly increased frequency of C allele [53]. This polymorphism impacts the hyperandrogenic phenotype in women with PCOS [53, 65, 81]. Interestingly, this polymorphism negatively influenced metabolic traits including obesity [80, 82], waist circumference [80], and insulin resistance [80]. A meticulous meta-analysis taking into consideration all studies revealed that this variant was not associated with risk of PCOS development when considering any genetic model or even after stratification by country and ethnicity. Furthermore, amongst studies which were in Hardy-Weinberg equilibrium, significantly increased risk was seen considering the dominant genetic model. However, they suggest sample size may also influence these associations as shown by increased risk in small sample compared to large sample studies [85].

*CYP19 Gene*. The aromatase p450 enzyme, essential for synthesis of estrogen from androgens, is encoded by* CYP19* gene on chromosome 15q21.2 [86]. Reduced aromatase activity in both lean and obese women with PCOS has reported [87] and activity is further inhibited by hyperandrogenemia [28]. Yang et al. have demonstrated decreased aromatase expression concomitant with increased levels of testosterone in follicular fluid derived from PCOS women [88]. Promoter hypermethylation and reduced CYP19A1 mRNA and protein levels were evident in PCOS ovaries, suggesting repressed aromatase expression [89]. An intronic variant rs2414096 was shown to be significantly associated with increased risk of PCOS development and with increased estradiol to testosterone ratio (E2/T), FSH levels, and age of menarche in Han Chinese women [90]. Raised PCOS symptom score and changes in circulating estradiol and testosterone concentrations were observed in adolescent girls in the UK carrying this polymorphism [91]. Additionally, certain promoter variants were independently associated with PCOS symptom score in UK adolescents [92]. The rs2414096 polymorphism lacked association with PCOS or with alterations in hormonal and metabolic variables after undergoing a 6-month treatment regime of oral contraceptives in both anovulatory and ovulatory PCOS women [93]. Another common polymorphism, a tetranucleotide repeat polymorphism (TTTA)_n_ in the fourth intron related to suboptimal aromatase activity [94], has been investigated. Reports are available indicating that short allele repeats, predominantly consisting of seven repeats, are prevalent in Greek [94–96] and Han Chinese [97] women with PCOS compared to controls. These short repeat alleles were associated with hormonal parameters including increased testosterone levels, high LH : FSH ratios [94], and reduced reproductive markers such as number of large follicles and total oocyte count [95]. Interestingly, these alleles predicted successful pregnancy following assisted reproductive technique intervention [95]. Carriers of 11 repeat alleles are also commonly found in Chinese women with PCOS [97, 98] which influence lipid metabolism [97]. Another polymorphism, rs2470152, did not affect PCOS risk but the heterozygous TC genotype was found to be significantly associated with increased testosterone levels with decreased E2/T ratio, suggesting role of this polymorphism in regulating aromatase activity [99]. A missense polymorphism, Arg264Cys, increases aromatase activity and affects PCOS susceptibility [100]. However, the above findings indicate a definite role of this gene in PCOS outcome.

*AR Gene*. The androgen receptor (AR) gene located on the X chromosome encodes the AR, which consists of a poorly conserved N terminal domain containing highly polymorphic CAG repeats [101]. An inverse correlation has been demonstrated between CAG repeat number and AR transactivation efficiency [102]. An interesting case study reported that a woman carrying a heterozygous* AR* gene mutation gave birth to a baby with androgen insensitivity syndrome suggesting plausible repercussions on reproductive outcomes associated with* AR* gene mutations [103] and not only with repeat lengths. AR has been primarily localized in the theca interna cells of preantral follicles, granulosa cells of preantral and antral follicles, and both theca and granulosa cells of dominant follicles [104]. Inconsistent associations of the differences in number of CAG repeats in exon 1 have been reported with PCOS prevalence. It has later been ascertained that short* AR* CAG repeats were more frequent in PCOS cases and may possibly be linked to PCOS onset in both Chinese and Caucasian populations [100, 105–108]. This may contribute to the inherent hyperandrogenic phenotype commonly seen in women with PCOS by increasing AR activity and enhancing androgen sensitivity to even low circulating levels of testosterone, thereby promoting hirsutism, acne, and irregular cycles [106, 109]. Anovulatory normoandrogenic PCOS women showed a significant trend towards short CAG repeat length indicating increased intrinsic androgen sensitivity [110]. Furthermore, they found that Indian women showed comparatively shorter repeat lengths compared to Chinese women, indicating possible role of ethnic variation [110]. No association of* AR* CAG repeat lengths with PCOS was reported in Indian [111], Slovene [112], Korean [113], Croatian, [114] and Finnish women [115]. A few studies have indicated that CAG repeat lengths may also modify both testosterone and insulin resistance parameters in women with PCOS despite failing to show association with PCOS risk. This CAG repeat polymorphism was found to be a significant predictor of serum circulating testosterone levels in Croatian [114], Brazilian [116], Chinese [101] and Korean [113] women with PCOS. German women carrying short CAG repeats presented with increased testosterone which in turn aggravated insulin resistance in these women suggesting a putative effect of CAG repeats as an underlying mechanism of hyperandrogenemia induced insulin resistance [117]. In contrast, infertile Australian PCOS women showed preferential expression of long CAG repeat alleles compared to fertile PCOS women [118]. Meta-analyses examining the relationship between CAG repeat lengths at* AR *and PCOS risk have concluded that they may not be major determining factors in PCOS etiology [111, 119, 120]. Apart from CAG repeat, other groups have concluded that a GGN repeat polymorphism and rs6152G/A polymorphism were also significantly associated with PCOS in Chinese women [121, 122]. Thus,* AR* polymorphisms may exacerbate the hyperandrogenic phenotype of women with PCOS.

*SHBG Gene*. Sex hormone binding globulin (SHBG) is primarily synthesized in the liver, binds androgens, and estrogen with high affinity, thereby lowering circulating steroid hormones and rendering them biologically unavailable to target tissues [123]. Several polymorphisms in the* SHBG* gene located on chromosome 17 have been shown to alter hepatic biosynthesis, plasma levels, and plasma clearance efficiency of SHBG, thereby regulating the distribution of sex steroid hormones [123]. Two novel coding region mutations were discovered in a woman showing severe SHBG deficiency, one which resulted in abnormal glycosylation and the other to truncated SHBG synthesis. This led to remarkably low SHBG levels with elevated circulating free testosterone concentrations [124]. The putative genetic contribution of* SHBG* polymorphisms was further supported by evidence of association of longer TAAAA repeats with late onset of menarche [125] and decreased SHBG levels in hirsute French women [126]. Long TAAAA repeat alleles failed to show association with PCOS risk in Croatian [127], Slovenian [128], French [126], and Chinese [129] women. However Greek women with PCOS had significantly greater frequency of long repeat alleles compared to controls [130]. An inverse association between TAAAA repeat polymorphism alone [126–128, 130] or coupled with short* AR* CAG repeats [96] and SHBG serum levels has been established. Greek women with PCOS having long* SHBG* alleles coupled with short* CYP19* alleles demonstrated low SHBG levels and increased testosterone levels with raised FAI, DHEAS and T/E2 ratios [96]. A meta-analysis was unable to draw a conclusive association between the TAAAA repeat polymorphism with PCOS risk indicating that it may not be a reliable predictor of PCOS onset [131]. A functional missense polymorphism in exon 8 causes an amino acid change from aspartic acid to asparagine (D327N), delays SHBG half-life, and influences the metabolism of SHBG [126]. Another missense polymorphism, E326K lowered SHBG levels in women with PCOS independently of BMI, androgen, and insulin related traits [132]. Family-based and case-control association studies have found that rs1799941 and rs727428 in* SHBG* gene influenced SHBG metabolism in American and Mediterranean women with PCOS [133, 134], but not PCOS risk [133]. A recent study in Bahraini women has concluded that haplotypes spanning six polymorphisms were associated with either increased or decreased PCOS susceptibility [135] rekindling interest in* SHBG* gene polymorphisms in PCOS susceptibility.

*StAR Gene*. The* StAR* gene located on chromosome 8p11.2 encodes the steroidogenic acute regulatory protein which binds to and facilitates uptake of cholesterol into mitochondria of cells for steroidogenesis. However a pilot study carried out in Iranian women investigating seven known polymorphisms showed no significant association with PCOS risk [136].

*HSD17B5 Gene*. The enzyme type 17*β*-hydroxysteroid dehydrogenase type 5 (HSD17B5) is instrumental in converting androstenedione to testosterone in theca cells and adrenal glands [137]. The −71A/G polymorphism in the promoter region was revealed for the first time by Qin et al., who also investigated its prevalence in a population of ethnically diverse PCOS women. Here they found that this variant was associated with PCOS susceptibility in Caucasian but not in African American women with PCOS [138]. It also modulates testosterone biosynthesis and thereby plasma testosterone levels [138]. Subsequent studies failed to find this association in Greek [139] and Caucasian [140] women with PCOS. Intronic polymorphism rs12529 affected testosterone levels but PCOS risk remained unchanged in Chinese women. On the other hand, rs1937845 not only increased risk of PCOS development but also increased homeostasis model assessment of *β*-cell function (HOMA-B) index and testosterone levels in these women [137]. In Brazilian women with PCOS, improvement in hyperandrogenic phenotype could be attributed to treatment regimen with oral contraceptive pills but not* HSD17B5* polymorphisms [141].

*INSL3 Gene*. Insulin-like factor 3 (INSL3) is localized in the thecal cells and corpus luteum of the ovary. A pioneering study by Glister et al. established the role of INSL3-RXFP2 signaling in maintaining androgen production by the ovarian theca cells [142]. Recently, women with PCOS were reported to have increased serum INSL3 levels [143–145].* INSL3 *polymorphisms may have an important role in modulating ovarian steroidogenesis and hence contribute to the pathogenesis of PCOS. To the best of our knowledge, our group has conducted the first case-control association study investigating relationship between* INSL3 *polymorphisms and its haplotypes with PCOS susceptibility and its related traits in a well characterized cohort of Indian women with PCOS [146]. Our study showed that the A/G* rs6523 *polymorphism present in exon 1 of* INSL3 *was significantly associated with PCOS susceptibility. Other coding region polymorphisms along with the* rs6543 *SNP affect both the metabolic and hyperandrogenemia related traits of PCOS in both controls and women with PCOS. These polymorphisms have differential influence depending on the physiological state present [146]. No other studies have been attempted to replicate this association till date.

## 5. Conclusion

PCOS remains an endocrine enigma even today characterized by adverse hormonal perturbations raising metabolic and gynecological concerns in affected women. Genetic factors work in tandem with environmental signals contributing to its pathogenesis. A hallmark feature of PCOS remains augmented androgen synthesis and consequent circulating levels which is frequently associated with cosmetic complaints including hirsutism, acne, and alopecia. The ovary remains the primary source of hyperandrogenism in women with PCOS. Thecal cell hyperplasia coupled with enhanced steroidogenic potential of androgen pathway enzymes may contribute to excess androgen production in ovaries of affected women. The current review has encapsulated salient findings from candidate gene based association studies of polymorphisms in genes involved in steroidogenesis as well as androgen levels and action which are presumed to govern PCOS susceptibility and phenotypic heterogeneity of the disorder. However, candidate gene studies have not provided conclusive results due to different diagnostic criteria, the likely contribution of multiple genes, differences in lifestyle, environmental factors, and the sample size studied. On the other hand, genome-wide association studies (GWAS) empower researchers with the capacity to explore thousands of variants across the entire genome in both case and control participants to uncover association of genetic variants with complex disease in an unbiased manner. The notable GWAS studies in Chinese populations have essentially offered several loci mapping to* DENND1A*,* THADA*,* LHCGR*,* FSHR*,* INSR*,* TOX3*,* YAP1*,* RAB5B*,* c9orf3*,* HMGA2*, and* SUMO1P1/ZNF217* involved in steroidogenesis, gonadotropin action and regulation, follicular development, insulin signaling and type 2 diabetes mellitus (T2DM), calcium signaling, and endocytosis [147, 148]. Of these loci,* DENND1A* has been implicated as a driving force for PCOS hyperandrogenemia. Overexpression in normal ovaries was found to upregulate ovarian steroidogenesis whereas knockdown decreases steroid synthesis by reducing transcription of CYP11A1 and CYP17 [149]. Interestingly, alternative splicing of DENND1A to produce v.2 variant is supposed to be important in PCOS development [150] and DENND1A v.2 was highly concentrated in theca cells of ovaries of women with PCOS [149]. On the other hand, although DENDD1A v.1 was abundant in the NCI-H295 adrenal steroidogenic cell line, overexpression of v.2 increased expression of CYP17 and CYP11A enzymes [150]. The association of the* LHCGR* locus with PCOS in GWAS [148] strengthens the rationale that alteration in receptor expression could contribute to LH hyperstimulation, thereby enhancing steroidogenesis. Findings from GWAS in European population has highlighted the significant association of gene polymorphisms in* FDFT1 *and* GATA4 *involved in cholesterol synthesis and a potent regulator of steroidogenic gene transcription, respectively, suggesting altered androgen synthesis [151]. Thus while candidate genes have offered substantial evidence to strengthen the role of genetic variants in modulating PCOS hyperandrogenism, GWAS has provided new clues which need to be explored in greater detail in different ethnic populations. Selection of suitable candidate genes should continue in order to successfully delineate the genetic underpinnings of a multigenic complex disorder like PCOS. These would pave the way for establishing genetic predisposition profiles which could be harnessed for designing therapeutic management strategies in future.
